# 4395 alpha-Synuclein Induced Reactive Gliosis

**DOI:** 10.1017/cts.2020.52

**Published:** 2020-07-29

**Authors:** Sean David Carey, Sarah Alshawi, Mondona McCann, Kathleen Maguire-Zeiss

**Affiliations:** 1 Georgetown - Howard Universities; 2 Georgetown University, Department of Biology; 3 GUMC, Interdisciplinary Program in Neuroscience; 4 GUMC, Department of Neuroscience

## Abstract

OBJECTIVES/GOALS: Reactive gliosis is a hallmark of neurodegenerative disease and is characterized by the release of pro-inflammatory cytokines and physiologic changes to glial cells. Our work identifies a novel inflammatory glial-glial cell interaction and role for mGluR5 that has the potential to provide novel insight into the mechanisms of neurodegeneration. METHODS/STUDY POPULATION: **Cell Culture:** Mouse primary astrocytes and microglia were isolated from P0-P3 C57BL/6 or Cx3cr1^GFP/+^ mice.^1^ **Treatment:** Glia were treated with oligomeric α-synuclein 1μg/mL or mGluR5 agonist CHPG 100 μM.^2,3^ **ELISA:** Glia culture media was collected and analyzed according to the manufacturer. **qRT-PCR:** TaqMan™ probes were used according to manufacturer on extracted glia mRNA. **ICC:** Microglia were labeled with 1:750 Rb x Iba1 (Wako) and 1:500 Alexa Fluor 488 Gt x Rb. **Phagocytosis Assay:** Primary glia were treated with α-synuclein or astrocyte-conditioned culture media for 24-48hrs. For treatment of microglia with conditioned media, astrocytes were washed with PBS and fresh media was added to prevent carry over of α-synuclein to microglia. The number of fluorescent microbeads per microglia was quantified. RESULTS/ANTICIPATED RESULTS: Mouse primary cortical astrocytes simulated with α-synuclein aggregates adopt a reactive A1 phenotype independent of microglial stimulation. This A1 phenotype is characterized by release of pro-inflammatory cytokines including Complement Component 3 and the monocyte chemoattractant CCL2. Reactive astrocyte media induces a phagocytic phenotype in primary mouse microglia. Along with this, α-synuclein-directed microglial phagocytosis was attenuated with the addition of the mGluR5 agonist CHPG. DISCUSSION/SIGNIFICANCE OF IMPACT: Our findings suggest that oligomeric α-synuclein is capable of inducing a reactive phenotype in astrocytes independent of microglia and implicate crosstalk between glia as an important mediator of inflammation and microglial phagocytosis in synucleinopathies.

